# Correction: A prebiotic diet modulates microglial states and motor deficits in α-synuclein overexpressing mice

**DOI:** 10.7554/eLife.92367

**Published:** 2023-08-31

**Authors:** Reem Abdel-Haq, Johannes CM Schlachetzki, Joseph C Boktor, Thaisa M Cantu-Jungles, Taren Thron, Mengying Zhang, John W Bostick, Tahmineh Khazaei, Sujatha Chilakala, Livia H Morais, Greg Humphrey, Ali Keshavarzian, Jonathan E Katz, Matthew Thomson, Rob Knight, Viviana Gradinaru, Bruce R Hamaker, Christopher K Glass, Sarkis K Mazmanian

**Keywords:** Mouse

Abdel-Haq R, Schlachetzki JCM, Boktor JC, Cantu-Jungles TM, Thron T, Zhang M, Bostick JW, Khazaei T, Chilakala S, Morais LH, Humphrey G, Keshavarzian A, Katz JE, Thomson M, Knight R, Gradinaru V, Hamaker BR, Glass CK, Mazmanian SK. 2022. A prebiotic diet modulates microglial states and motor deficits in α-synuclein overexpressing mice. *eLife* **11**:e81453. doi: 10.7554/eLife.81453.

Published 8 November 2022

In the original version of this paper, the plot for the adhesive removal test in Figure 1–figure supplement 2 panel D was mistakenly duplicated in panel A (beam traversal -- time to cross). This error occurred during figure preparation for submission. The associated source data, text, and remainder of the Figure are correct, and the interpretation of the results are unaffected.

The corrected Figure 1–figure supplement 2 is shown here, with panel A being updated:

**Figure 1:**
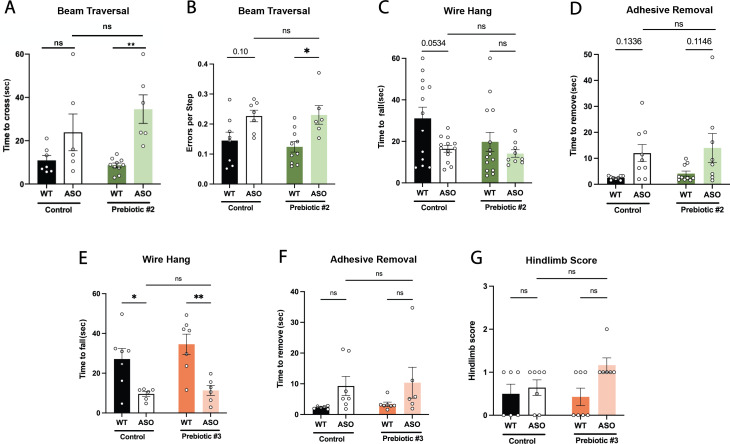


The originally published Figure 1–figure supplement 2 is shown for reference:

**Figure 2:**
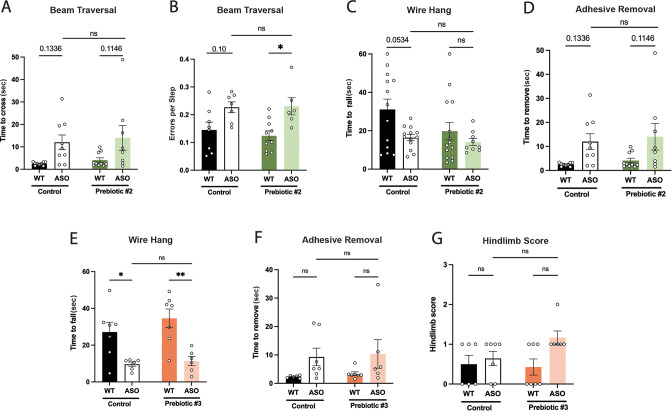


The article has been corrected accordingly.

