# Supplementary figures and images for: A prebiotic diet modulates microglial states and motor deficits in α-synuclein overexpressing mice
Source: eLife. 2022 Nov 8;11:e81453. doi: 10.7554/eLife.81453 (PMC9668333; doi:10.7554/eLife.81453)

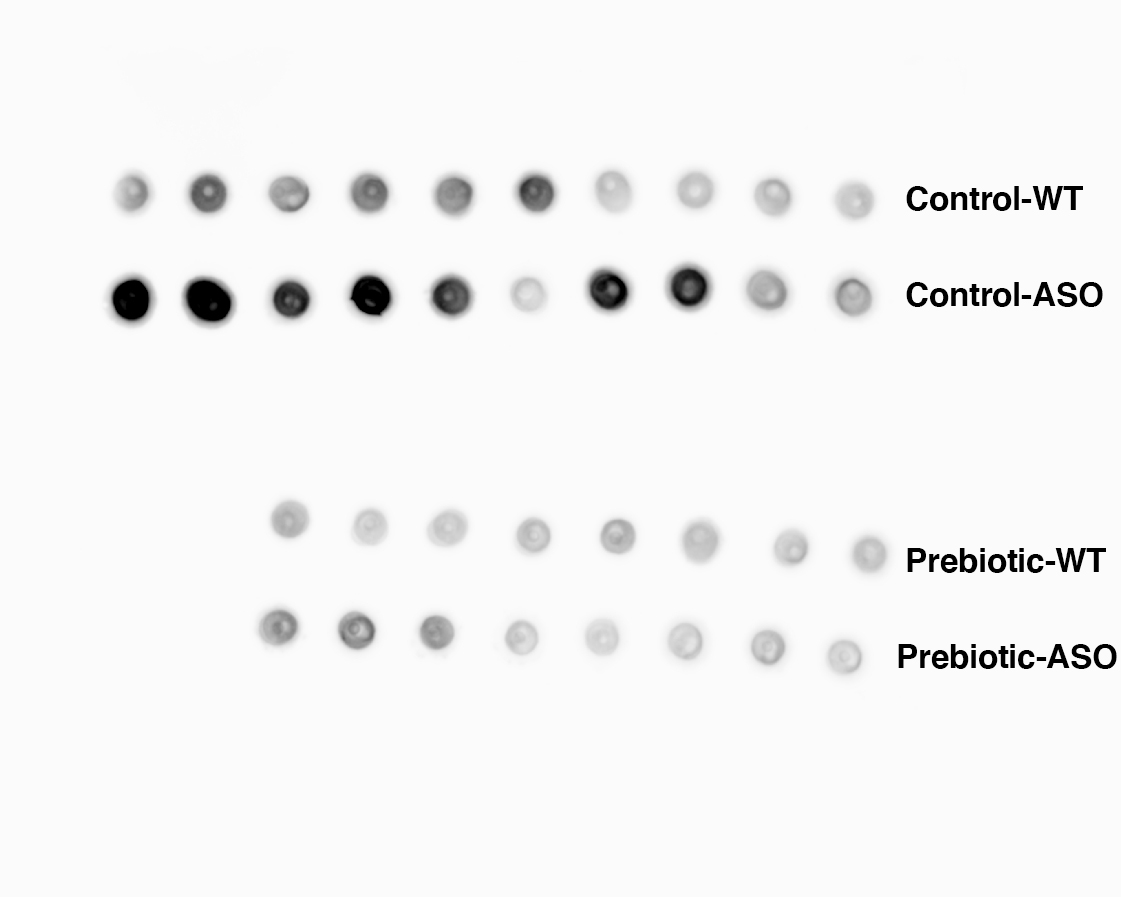

Supplement: Figure 1—source data 1. [file elife-81453-fig1-data1.zip › Figure 1-source data 1/Figure 1-source data 1-annotated.jpg]

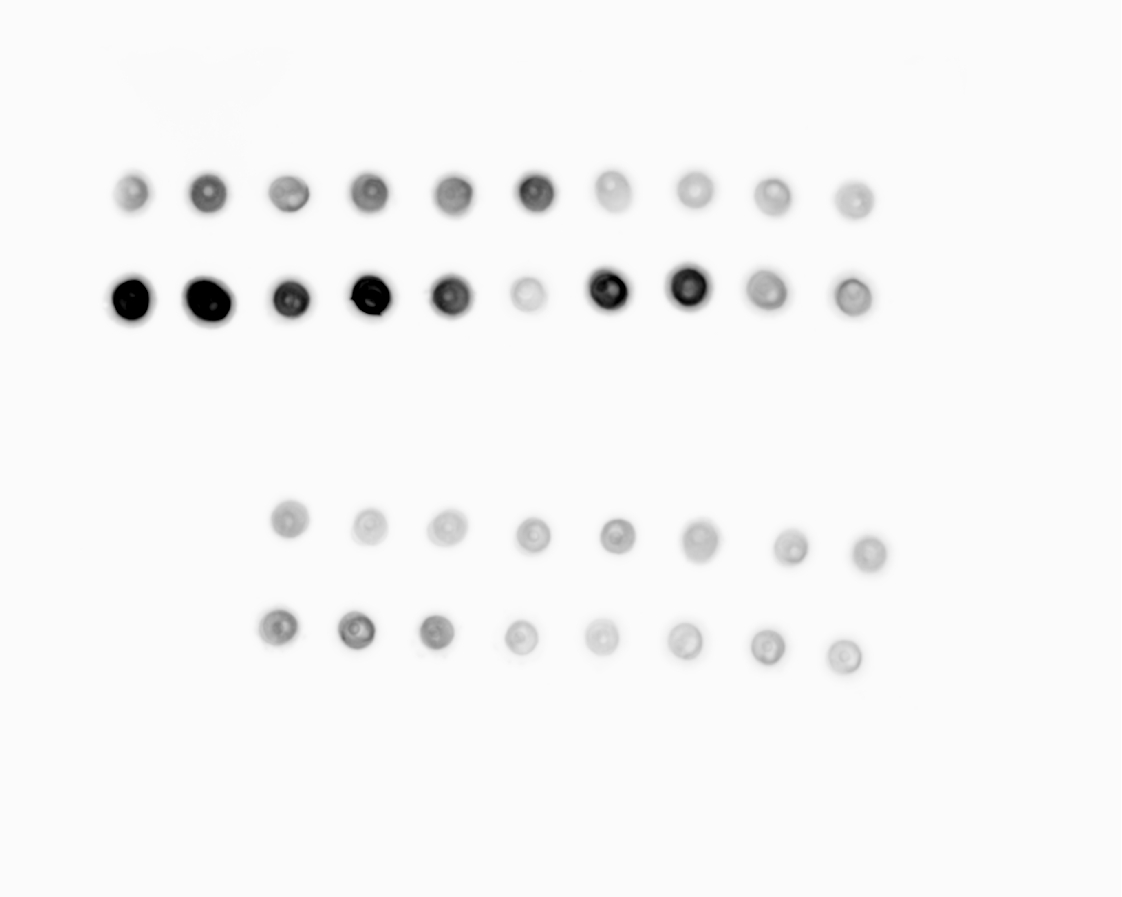

Supplement: Figure 1—source data 1. [file elife-81453-fig1-data1.zip › Figure 1-source data 1/Figure 1-source data 1.jpg]

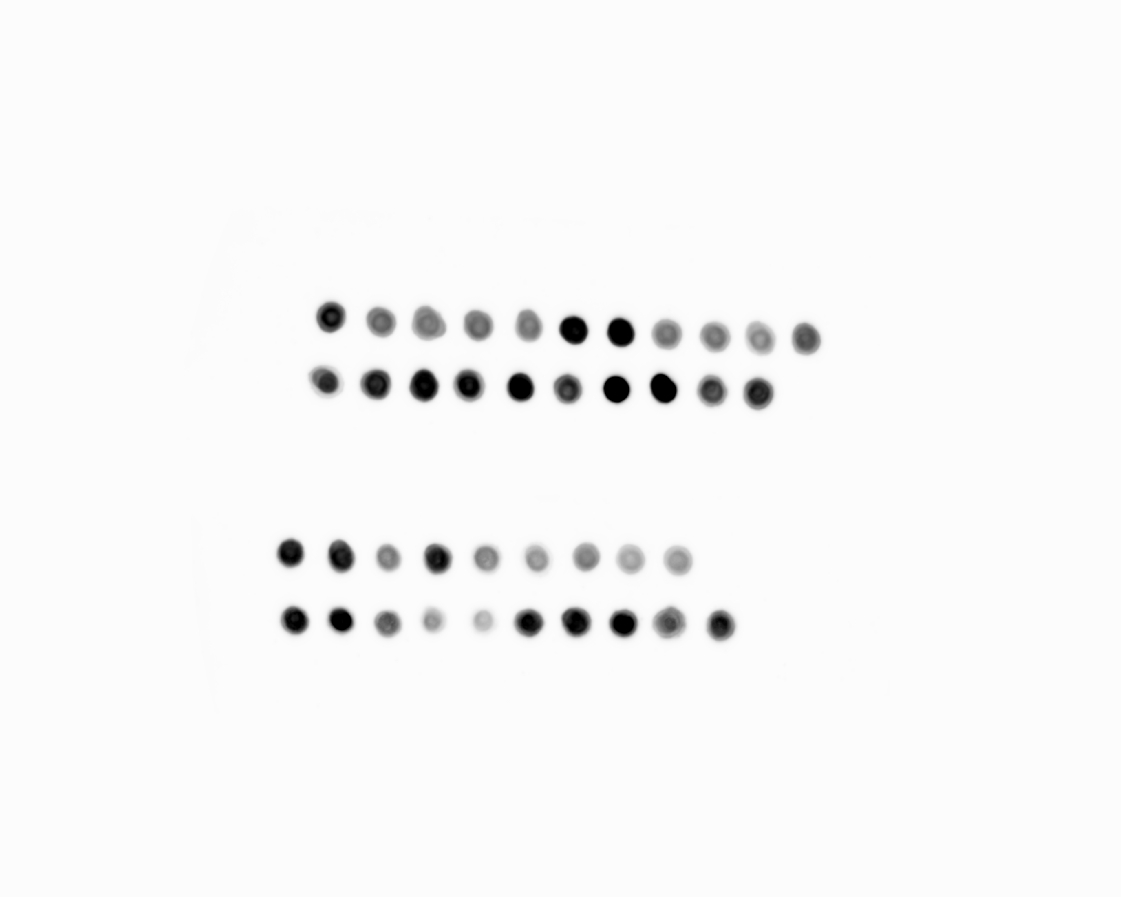

Supplement: Figure 1—source data 2. [file elife-81453-fig1-data2.zip › Figure 1-source data 2/Figure 1-source data 2.jpg]

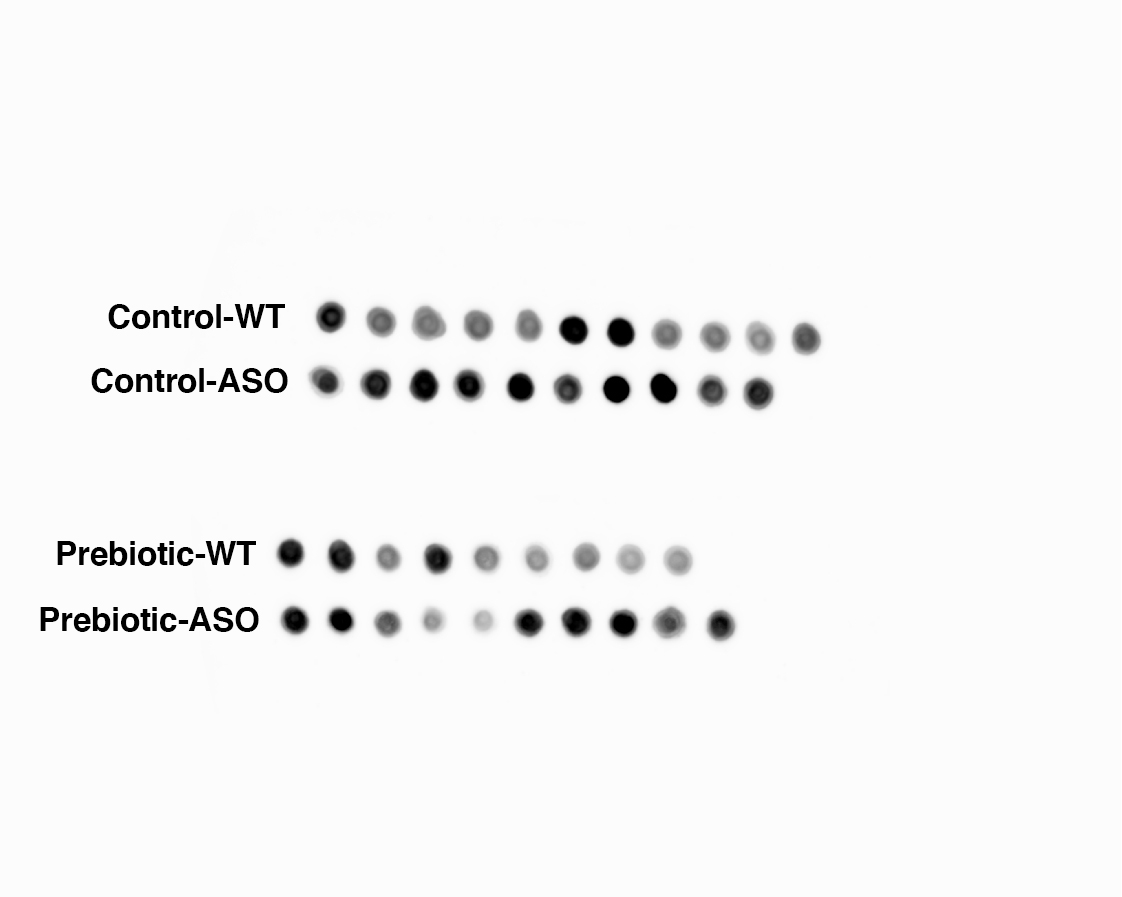

Supplement: Figure 1—source data 2. [file elife-81453-fig1-data2.zip › Figure 1-source data 2/Figure 1-source data 2-annotated.jpg]

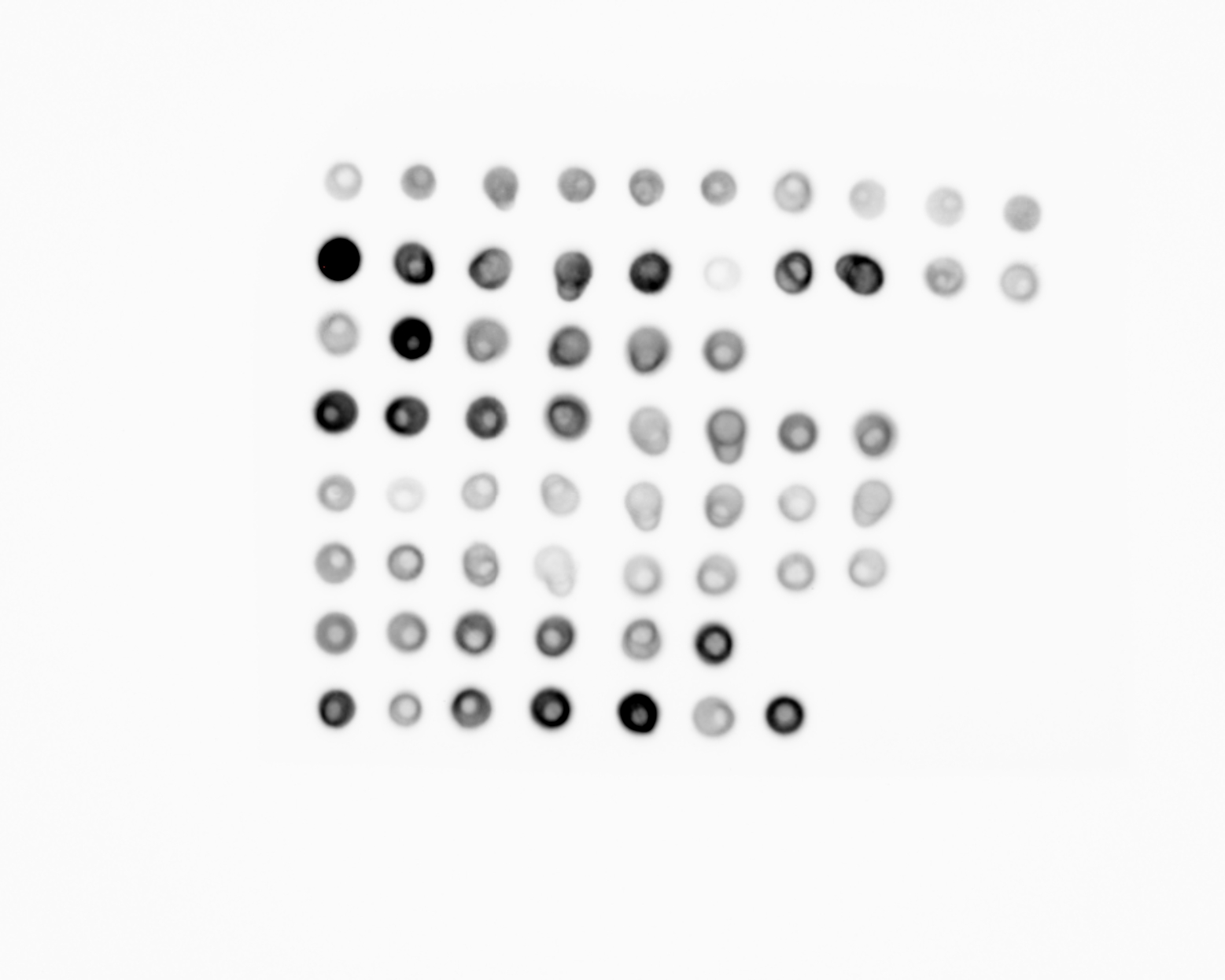

Supplement: Figure 5—source data 1. [file elife-81453-fig5-data1.zip › Figure 5-source data 1/Figure 5-source data 1.jpg]

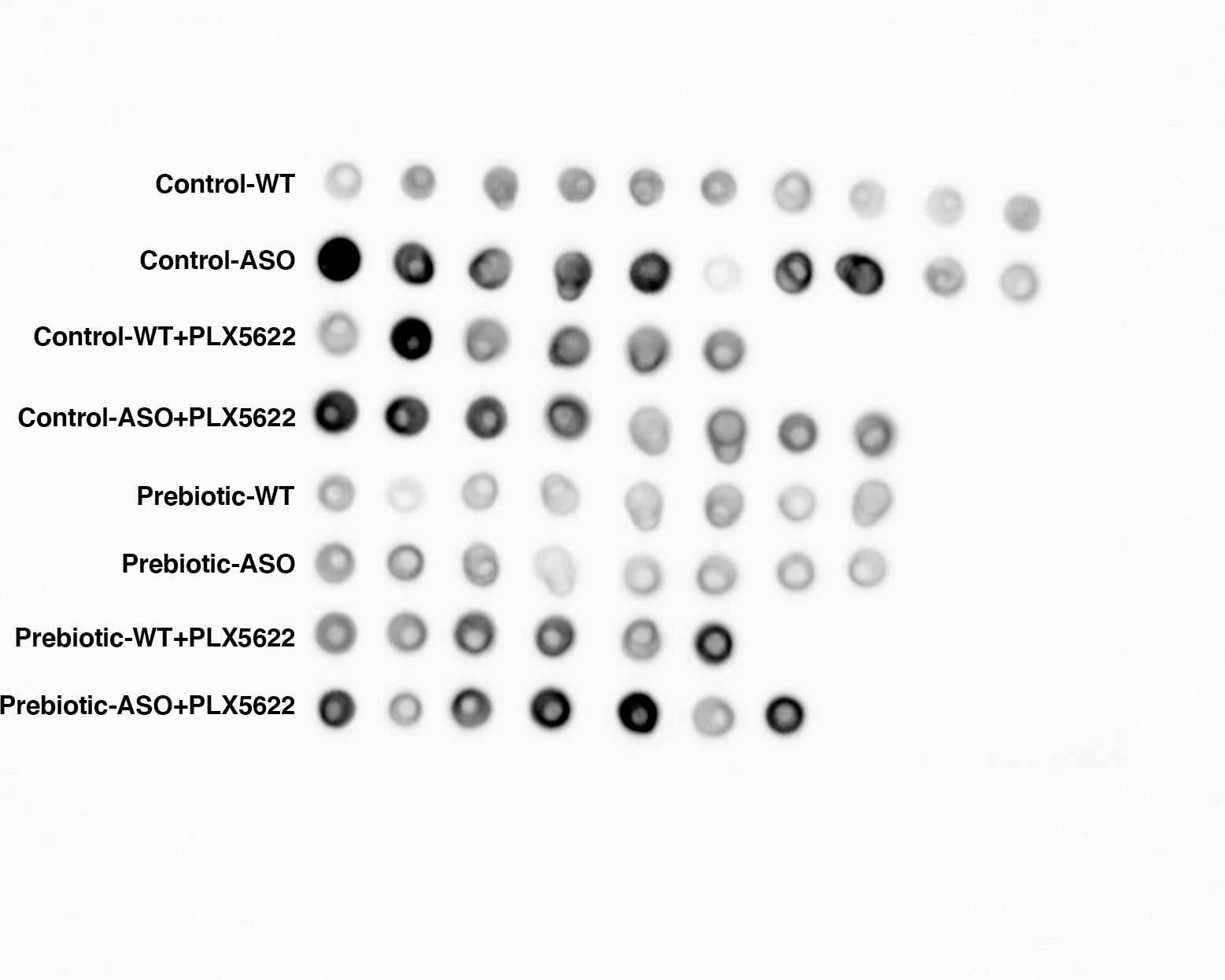

Supplement: Figure 5—source data 1. [file elife-81453-fig5-data1.zip › Figure 5-source data 1/Figure 5-source data 1-annotated.jpg]

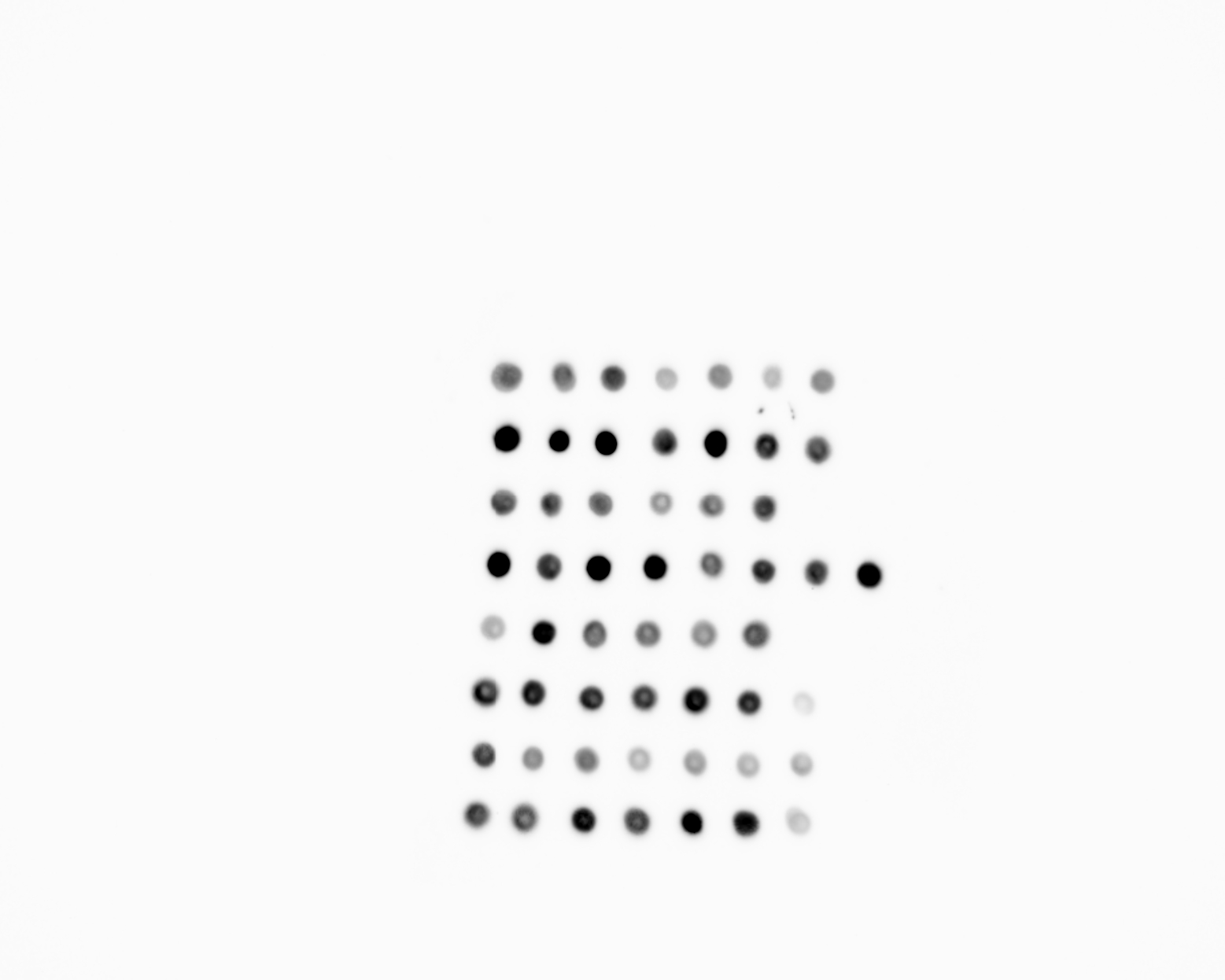

Supplement: Figure 5—source data 2. [file elife-81453-fig5-data2.zip › Figure 5-source data 2/Figure 5-source data 2.jpg]

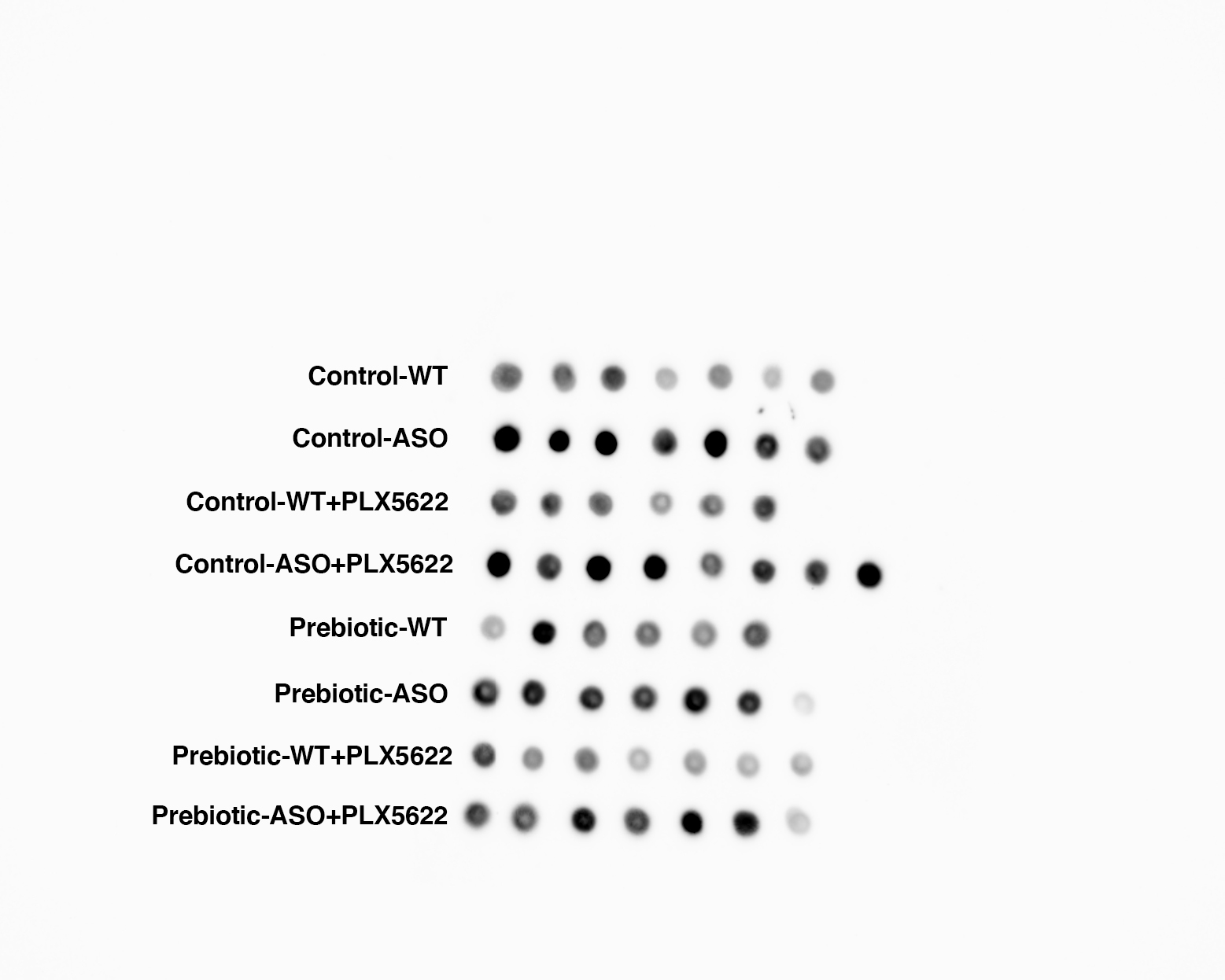

Supplement: Figure 5—source data 2. [file elife-81453-fig5-data2.zip › Figure 5-source data 2/Figure 5-source data 2-annotated.jpg]
